# Family doctor contract services and health-related quality of life among patients with chronic diseases in rural China: what is the role of socioeconomic status?

**DOI:** 10.1186/s12939-021-01530-2

**Published:** 2021-08-26

**Authors:** Zhixian Li, Jie Li, Peipei Fu, Yan Chen, Zhengyue Jing, Yemin Yuan, Shijun Yang, Chen Yan, Wenjuan Li, Jie Li, Zhen Gui, Chengchao Zhou

**Affiliations:** 1 Centre for Health Management and Policy Research, School of Public Health, Cheeloo College of Medicine, Shandong University, Jinan, 250012 China; 2 NHC Key Lab of Health Economics and Policy Research, Shandong University, Jinan, 250012 China; 3 School of Public Health, Wannan Medical College, Wuhu, 241002 China

**Keywords:** Family doctor contract services, Health quality of life, EQ-5D, Socioeconomic status

## Abstract

**Purpose:**

Few studies explored the relationship between the family doctor contract services (FDCS) and health-related quality of life (HRQOL) among patients with chronic diseases in rural China. This study aims to explore the relationship between the status of signing on FDCS and HRQOL among patients with chronic diseases and examine whether there are differences in the relationship between different socioeconomic status (SES).

**Methods:**

A total of 1,210 respondents were included in this study. HRQOL was measured by EQ-5D-3L. The contracting status was divided into uncontracted and contracted. Tobit regression and Logistic regression were employed to explore the association between contracting status and HRQOL. The interaction terms were included to explore the differences in the association among different SES.

**Results:**

Contracting with family doctors was associated with HRQOL (coefficient = 0.042; 95%CI 0.008 to 0.075). The association was different among different socioeconomic levels that the contracting status was only associated with HRQOL in sub-high-income (*P* < 0.01) and highly educated patients (*P* < 0.05). Compared with uncontracted patients, contracted patients reported higher ED-5D-3L utility value in the sub-high-income group (coefficient = 0.078; 95%CI 0.017 to 0.140) and high educational attainment (coefficient = 0.266; 95%CI 0.119 to 0.413).

**Conclusions:**

This study found a significant association between FDCS and HRQOL among chronic patients in rural Shandong, China. This relationship varied by income levels and educational attainment. The government should take efforts to formulate a variety of measures to encourage chronic patients to contract with family doctors, with special attention to people with low SES.

**Supplementary Information:**

The online version contains supplementary material available at 10.1186/s12939-021-01530-2.

## Introduction

With the aging of the population and the change of disease spectrum, the morbidity and mortality of chronic diseases such as hypertension, diabetes, and coronary heart disease has been on a rapid rise in the past decades. According to the World Health Statistics Report 2020, 42 million people worldwide died of chronic diseases in 2016, accounting for about 71% of all deaths [1]. In China, chronic diseases account for more than 80% of the total 10.3 million deaths and over 70% of the burden of disease each year [2, 3]. Previous studies showed that grass-root preventive interventions could improve the unhealthy lifestyles of people with chronic diseases. Primary health care can effectively reduce the morbidity and mortality of chronic diseases, control the condition of those with chronic diseases, and reduce the burden of chronic diseases [4, 5]. Therefore, over 50 countries have established the gatekeeper system in health care, in which the primary care physicians played a key role [6].

Due to different national conditions, there exist differences in the specific service mode and operation mechanism in the family doctor service system. The family doctor system originated in the United States in the 1960s, which incorporated health management into the community general practitioner service model, and conducted active follow-up observation on patients with chronic diseases [7, 8]. In Britain, the National Health Service, established in 1948, adopted the national management model that required citizens to contract with their family doctors [9]. General practitioners in Germany are freely chosen to provide their services to contracted residents [10]. As a developing country, China has been in the process of establishing family doctor contract services (FDCS) system in health care since 2009. The long-term medical cooperation between family doctors and the residents is established by signing the contract of the service, so as to better understand the healthcare needs and improve the health status of the residents through the formulation of personalized intervention plans [11, 12]. In 2016, the Chinese government fully implemented the family doctor signing system. Due to the high incidence rate and high burden of chronic diseases, patients with chronic diseases were classified as the priority population [13]. In 2017, the coverage rate of FDCS for the priority population was required to reach over 60%. By 2020, the goal of full coverage of the FDCS system will be basically achieved. The team providing contract services usually consists of general practitioners, nurses, and public health doctors. In Shandong province, family doctors carry out work according to the Guide for Shandong Province Family Doctor Contracting Service, and residents and family doctor teams signed in a voluntary way. Family doctors provide residents who have signed with basic medical services and public health services. In terms of basic medical services, family doctors provide services packages including common diseases treatment, follow-ups, and referrals. In terms of public health services, family doctors conduct proactive life intervention measures to prevent and manage chronic conditions, create and manage individual health records, and give annual physical health examinations for the contracted residents.

Health-related quality of life (HRQOL) is a multidimensional indicator of health that includes physical functioning, mental health, and the socially relevant roles that an individual perceives over time [14]. It is often used to support public health and health policy and has become very important at the policy level [15]. A study in Norway demonstrated that family doctors can improve health-related quality of life in older patients exposed to polypharmacy [16]. Unfortunately, in China, the association between FDCS and HRQOL among patients with chronic diseases has not been extensively studied.

Socioeconomic status (SES) depends on a combination of variables including occupation, education, income, and place of residence [17]. Studies indicated chronic disease patients with low SES have lower HRQOL [18], and there were differences in income levels in terms of the relationship of FDCS and the utilization of health services for patients with chronic diseases [19]. Other studies have demonstrated there was a differential association between chronic conditions and HRQOL across different SES positions [20]. These findings suggest the association between FDCS and HRQOL might be different among patients of chronic diseases with different SES, which needs to be clarified.

However, to date, there are also few studies examined the possible difference in the association between FDCS and HRQOL. Thus, the present study aims to explore the relationship between FDCS and HRQOL among patients with chronic diseases and examine whether there are differences in this association across different SES. Based on the previous findings, we hypothesized that (1) contracting with a family doctor was associated with better HRQOL; (2) there were differences in the association between FDCS and HRQOL in different SES.

## Methods

### Study population

The data used in this study were from the survey on FDCS conducted in Shandong province, China in 2018. Shandong is the second-most populous province in China with more than 100 million population in 2018, of which about 40% lived in rural areas. The multi-stage random sampling method was used to select the subjects. Firstly, we randomly selected three cities (Liaocheng, Zibo, and Binzhou) as study sites. Secondly, two counties were randomly selected from each sample city. Thirdly, five townships were randomly selected from each country and then six villages from each selected township were randomly selected. Finally, 16 households were randomly selected from each sample village. If there were two or more eligible rural residents from one family, only one was selected to participate in the survey (generally the head of a household, few are non-heads of households, see Supplementary Table 1). In rural China, generally speaking, the head of the household or his spouse knows the household income best. Strict random number table sampling was used throughout the study. A total of 2,979 respondents were interviewed, of whom 1,210 patients with chronic diseases (hypertension, coronary heart disease, or diabetes) were included in the analysis.

The investigators were recruited from Shandong University. They were strictly trained before the investigation, including understanding the principles and methods of the survey, and standardizing the definition and interview skills of each study indicator, with the purpose of ensuring the quality of the survey. After the training, the investigators were given the test on training effectiveness, and only those who were qualified could participate in the formal investigation. Each sampled township was also supervised by a trained supervisor who was responsible for guiding and (logical) checking survey questionnaires to ensure the accuracy and completeness of the questionnaires.

### Measures

#### Family doctor contract services

The status of FDCS was a two-category variable. It was measured by the following yes–no question: “Did you contract with the family doctors this year?” [21, 22], which could be respond with “yes” (scored as 1) or “no” (scored as 0).

#### Health-related quality of life

Health-related quality of life (HRQOL) was assessed using the EQ-5D questionnaire, which consists of 5 health dimensions (mobility, self-care, usual activities, pain/discomfort, and anxiety/depression). Each of the five dimensions has three levels indicating no problems, moderate problems, or severe problems [23]. The EQ-5D-3L utility values are generated by weighting each dimension of the HRQOL, which uses the time trade-off model set for the Chinese general population [24], ranging from -0.149 to 1.0, and the higher EQ-5D-3L utility values represent higher HRQOL.

### Socioeconomic status

The use of household income, employment status and education has been widely studied and validated as some of the most accurate indicators of SES [25–27]. In this study, SES was measured by educational attainment, employment status, and household income per capita reported by the participants. Educational attainment was recoded into three categories: low (primary education or below), intermediate (junior education), and high (senior education or above). Household income was collected by asking the respondents to report specific components, such as production income, wage income, transfer income, and others. Then the respondents were also asked to report the total annual household income for validation of the sum of the components. According to the quartile methods, household income per capita was classified into four categories: Q1, Q2, Q3, and Q4, from lowest to highest. We recoded employment status into two categories, unemployed and employed situation. Specifically, lower educational attainment, lower quartile, and unemployed status represent lower SES.

### Covariate variable

The marital status was divided into three categories: single, married, and others (divorced or widowed). The number of family members was classified into four categories: single, two members, three members, and four members or more. The drinking status was divided into three categories, including never drinkers, current drinkers, and former drinkers. Other covariate variables include age, gender, multiple chronic diseases status, and physical exercise.

### Statistical analyses

Statistical analyses were performed using Stata 14.0. The reported credible intervals (CIs) were calculated at the 95% level and *P* values less than 0.05 were considered statistically significant. First, we used frequencies and percentages to describe the demographic characteristics of the respondents by family doctors contracting status. Second, independent-samples t-test was used to compare the EQ-5D-3L utility values between contracting status. Third, Tobit regression was employed to explore the association between the contracting status of family doctors and EQ-5D-3L utility values. Logistic regression was performed to explore the association between contracting status and each dimension of EQ-5D-3L. All regression analyses were performed using the enter method [16]. In order to explore whether there were differences in the association among different SES (income, educational attainment, and employment status), interaction terms between contracting status and SES were introduced in the Tobit regression models, and if the interaction terms were statistically significant in that Tobit regression model, we further stratified the regression analyze by different SES.

## Results

### Socio-demographic characteristics

Table 1 shows the demographic characteristics of the respondents. Of 1,210 patients with chronic diseases, 29.3% contracted with family doctors. The majority of the patients were never drinkers (63.9%), female (55.9%), married (84.1%), and with two family members (49.9%), participate in physical activities (55%), and without multiple chronic diseases (70.7%). In terms of SES, the vast majority of respondents were employed (69.3%), had an education level of primary school or below (70.0%), and had the lowest income level (26.3%).

**Table 1:** Basic characteristics of FDCS among patients with chronic diseases in rural Shandong, China, 2018

Characteristic	N (%)	Contracting status		*P*-value
		**Yes (%)**	**No (%)**	
**Observations(%)**	1210 (100%)	355 (29.3)	855 (70.7)	
**Age (Years)**				0.395
< 50	118 (9.75)	41 (34.8)	77 (65.2)	
50–59	284 (23.47)	70 (24.6)	214 (75.4)	
60–69	504 (41.65)	143 (71.6)	361 (28.4)	
≥ 70	304 (25.12)	101 (66.8)	203 (33.2)	
**Gender**				0.003
Male	534 (44.1)	180 (33.7)	354 (66.3)	
Female	676 (55.9)	175 (25.9)	501 (74.1)	
**Educational attainment**				0.000
Primary education or below	798 (70.0)	205 (25.7)	593 (74.3)	
Junior education	298 (24.6)	100 (33.6)	198 (66.4)	
Senior education or above	114 (9.4)	50 (43.9)	64 (56.1)	
**Household income **^**a**^				0.002
Q1	318 (26.3)	74 (23.3)	244 (76.7)	
Q2	287 (23.7)	82 (28.6)	205 (71.4)	
Q3	303 (25.0)	95 (31.3)	208 (69.7)	
Q4	302 (25.0)	104 (34.4)	198 (65.6)	
**Multiple chronic diseases**				0.055
No	855 (70.7)	237 (27.7)	618 (72.3)	
Yes	355 (29.3)	118 (33.2)	237 (66.8)	
**Employment status**				0.058
Unemployed	372 (30.7)	123 (33.1)	249 (66.9)	
Employed	838 (69.3)	232 (27.7)	606 (72.3)	
**Drinking status**				0.000
Never drinkers	772 (63.9)	194 (25.1)	578 (74.9)	
Former drinkers	155 (12.8)	54 (34.8)	101 (65.2)	
Current drinkers	283 (23.3)	106 (37.6)	176 (62.4)	
**Physical exercise**				0.000
No	544 (45.0)	122 (22.4)	422 (77.6)	
Yes	666 (55.0)	233 (35.0)	433 (65.0)	
**Marital status**				0.336
Single	15 (1.2)	3 (20.0)	12 (80.0)	
Married	1017 (84.1)	307 (30.2)	710 (69.8)	
Others ^b^	178 (14.7)	45 (25.3)	133 (74.7)	
**Family members**				0.276
1	132 (10.9)	31 (23.5)	101 (76.5)	
2	604 (49.9)	177 (29.3)	427 (70.7)	
3	188 (15.6)	62 (33.0)	126 (67.0)	
≥ 4	286 (23.6)	85 (29.7)	201 (70.3)	

***Note***: *FDCS* Family doctor contract services

^a^ Q1 was the poorest and Q4 was the richest

^b^ Others include those who are divorced and widowed

### EQ-5D-3L values by different contracting status

As shown in Table 2, among the 1,210 respondents, the mean EQ-5D utility value was 0.844 (± 0.177). The independent-samples t-test results showed that there were significant differences in EQ-5D-3L utility value between different contracting status, the mean EQ-5D-3L utility values were higher among contracted patients than uncontracted patients, and more problems were reported in the uncontracted group of all dimensions.

**Table 2:** Observed utility values of EQ-5D-3L of chronic patients by contracting status and household income in rural Shandong, China, 2018

Characteristics	Mean ± SD ^a^	EQ-5D-3L (%) ^b^				
		**Mo**	**SC**	**UA**	**PD**	**AD**
**Total**	0.844 ± 0.177	24.9	11.9	24.5	52.5	22.3
**Contracting status **^**c**^						
No	0.831 ± 0.181	28.1	13.9	27.6	54.7	24.3
Yes	0.875 ± 0.141^***^	17.2	7.0	16.9	47.3	17.5
**Household income**						
**Q1**						
No	0.792 ± 0.195	34.8	19.7	35.3	61.9	31.6
Yes	0.849 ± 0.163^*^	24.3	10.8	27.0	50.0	20.3
**Q2**						
No	0.822 ± 0.185	30.7	15.6	25.4	56.6	23.9
Yes	0.873 ± 0.139^*^	14.6	7.3	18.3	48.8	19.5
**Q3**						
No	0.831 ± 0.177	27.4	13.5	30.3	57.2	23.6
Yes	0.889 ± 0.125^**^	15.8	5.3	13.7	45.3	12.6
**Q4**						
No	0.886 ± 0.144	17.7	5.6	17.7	41.4	16.7
Yes	0.883 ± 0.140	15.4	5.8	11.5	46.2	19.4
**Education attainment**						
**Primary education or below**						
No	0.809 ± 0.185	32.4	16.9	32.0	60.0	28.2
Yes	0.844 ± 0.149^*^	22.9	9.8	20.5	56.6	22.0
**Junior education**						
No	0.883 ± 0.153	18.2	6.6	16.7	42.9	13.6
Yes	0.904 ± 0.115	11.0	3.0	12.0	42.0	15.0
**Senior education or above**						
No	0.869 ± 0.181	18.8	9.4	20.3	43.8	21.9
Yes	0.942 ± 0.118^*^	6.0	4.0	12.0	20.0	4.0
**Employment status**						
**Unemployed**						
No	0.765 ± 0.204	42.6	24.1	41.8	68.6	29.7
Yes	0.842 ± 0.160^***^	30.1	12.2	23.6	51.2	21.9
**Employed**						
No	0.858 ± 0.162	22.1	9.7	21.8	49.0	22.1
Yes	0.893 ± 0.127^***^	10.3	4.3	13.4	45.3	15.1

***Note***: *HRQOL* Health-related quality of life, *MO* Mobility, *SC* Self-care, *UA* Usual activity, *PD* Pain/discomfort, *AD* Anxiety/depression

*P*-values with statistical significance: ^*^P < 0.05, ^**^P < 0.01, ^***^P < 0.001

^a^ Observed EQ-5D-3L utility values; SD: standard deviation

^b^ Observed frequency (%) of “have problems” in EQ-5D-3L dimensions

^c^ Independent-samples t-test was used to compare the EQ-5D utility values between contracting status

When stratified by household income, differences in EQ-5D-3L utility values were shown across different contracting statuses in low-income, sub-low-income and sub-high-income groups, whereas this difference was not found in high-income groups. When stratified by educational attainment, there were differences in EQ-5D-3L utility values across different contracting statuses in the primary school and below, high school and above age groups. Regardless of whether they were employed or not, the EQ-5D-3L utility values of different contracting statuses were significantly different. The EQ-5D-3L utility values of the contracted group was higher than that of the uncontracted group in all the groups which had significant differences.

### The relationship between family doctors contracting status and HRQOL among people with different SES

We present the results in three models so that the association between FDCS and HRQOL could be better explored (Table 3). In Model 1, after adjusting for the age, gender, marital status, family members, multiple chronic diseases status, drinking status and sports participation, the result demonstrated that FDCS was significantly associated with the HRQOL. Compared with those who did not contract with family doctors, the EQ-5D-3L utility (coefficient = 0.042; 95%CI 0.008 to 0.075) value of patients with chronic diseases who contracted with family doctors was significantly higher. When including the interaction terms between household income and contracting status into Tobit regression, model 2 shows an income difference in the association between contracting status and EQ-5D-3L utility value is significant (*P* < 0.05). Specifically, Table 4 shows that in the sub-high-income group, the EQ-5D-3L utility value of the contracted patients is higher than that of the non-contracted patients. When including the interaction terms between educational attainment and contracting status into Tobit regression, model 3 shows that there is also a significant difference in educational attainment between the contracting status and EQ-5D-3L utility value (*P* < 0.05). Among the highly educated group, contracted patients showed a significantly higher EQ-5D-3L utility value compared with non-contracted patients. In addition, the relationship between contracting status and HRQOL is not modified by employment situation. Furthermore, Table 4 shows that in the sub-high-income group, the contracted group reports fewer problems in mobility (*P* = 0.014; OR = 0.413), self-care (*P* = 0.033; OR = 0.310), and usual activity (*P* = 0.002; OR = 0.310) dimension of EQ-5D-3L. In the group with senior education or above, contracted patients report less problems in in mobility (*P* = 0.024; OR = 0.088), pain/discomfort (*P* = 0.002; OR = 0.136) and anxiety/depression (*P* = 0.006; OR = 0.050) dimension of EQ-5D-3L than uncontracted patients.

**Table 3:** The relationship between contracting status and HRQOL among chronic patients with different SES in rural Shandong, China, 2018

Characteristic	Model 1:no interaction			Model 2:Income × contracting status			Model 3:Educational attainment × contracting status		
	***P*-value**	**Coefficient**	**95%CI**	***P*-value**	**Coefficient**	**95%CI**	***P*-value**	**Coefficient**	**95%CI**
**Contracting status**									
No		Ref			Ref			Ref	
Yes	0.013	0.042	(0.008,0.075)	0.543	-0.020	(-0.083,0.044)	0.002	0.171	(0.064,0.277)
**Household income **^**a**^									
Q1		Ref			Ref			Ref	
Q2	0.796	0.005	(-0.036,0.047)	0.632	0.011	(-0.036,0.058)	0.762	0.006	(-0.035,0.047)
Q3	0.636	0.010	(-0.032,0.052)	0.714	0.009	(-0.039,0.057)	0.617	0.011	(-0.031,0.053)
Q4	0.005	0.062	(0.018,0.105)	0.000	0.090	(0.039,0.140)	0.005	0.063	(0.019,0.106)
**Educational attainment**									
Primary education or below		Ref			Ref			Ref	
Junior education	0.001	0.064	(0.026,0.102)	0.001	0.064	(0.026,0.102)	0.002	0.073	(0.028,0.117)
Senior education or above	0.007	0.079	(0.021,0.1)	0.006	0.080	(0.023,0.138)	0.461	0.027	(-0.044,0.098)
**Gender**									
Male		Ref			Ref			Ref	
Female	0.017	-0.054	(-0.097,-0.010)	0.023	-0.050	(-0.095,0.007)	0.014	-0.055	(-0.985,-0.011)
**Age**									
< 50		Ref			Ref			Ref	
50–59	0.175	-0.041	(-0.100,0.018)	0.171	-0.041	(-0.100,0.178)	0.236	-0.036	(-0.095,0.023)
60–69	0.071	-0.054	(-0.011,0.005)	0.065	-0.055	(-0.114,0.003)	0.092	-0.050	(-0.109,0.008)
≥ 70	0.507	-0.023	(-0.090,0.044)	0.470	-0.025	(-0.091,0.042)	0.595	-0.018	(-0.085,0.049)
**Multiple chronic diseases**									
No		Ref			Ref			Ref	
Yes	0.000	-0.067	(-0.099,-0.035)	0.000	-0.068	(-0.100,-0.036)	0.000	-0.069	(-0.101,-0.036)
**Employment status**									
Unemployed		Ref			Ref			Ref	
Employed	0.000	0.095	(0.061,0.129)	0.000	0.095	(0.060,0.129)	0.000	0.096	(0.062,0.131)
**Drinking status**									
Never drinkers		Ref			Ref			Ref	
Former drinkers	0.118	-0.043	(-0.097,0.011)	0.135	-0.041	(-0.095,0.013)	0.116	-0.043	(-0.097,0.011)
Current drinkers	0.299	0.025	(-0.022,0.073)	0.271	0.027	(-0.021,0.074)	0.269	0.026	(-0.021,0.074)
**Physical exercise**									
No		Ref			Ref			Ref	
Yes	0.000	-0.057	(-0.087,-0.027)	0.000	-0.057	(-0.087,-0.027)	0.000	-0.057	(-0.087,-0.027)
**Marital status**									
Single		Ref			Ref			Ref	
Married	0.259	0.077	(-0.057,0.211)	0.257	0.077	(-0.056,0.211)	0.270	0.075	(-0.058,0.209)
Others ^b^	0.591	0.036	(-0.100,0.170)	0.570	0.038	(-0.094,0.171)	0.579	0.037	(-0.095,0.170)
**Family members**									
1		Ref			Ref			Ref	Ref
2	0.623	-0.017	(-0.011,0.069)	0.633	-0.016	(-0.083,0.051)	0.755	-0.012	(-0.079,0.055)
3	0.966	0.002	(-0.013,0.072)	0.959	0.002	(-0.072,0.076)	0.897	0.005	(-0.069,0.079)
≥ 4	0.377	0.031		0.360	0.032	(-0.036,0.998)	0.349	0.033	(-0.036,0.101)
**Income × contracting status**									
Q1 × contracted				0.042	0.095	(0.004,0.187)			
Q2 × contracted				0.156	0.066	(-0.025,0.157)			
Q3 × contracted				0.052	0.088	(-0.001,0.177)			
**Educational attainment × contracting status**									
Primary education or below × contracted							0.020	-0.135	(-0.249,-0.022)
Junior education × contracted							0.011	-0.161	(-0.286,-0.038)

***Note:****HRQOL* Health-related quality of life, *SES* Socioeconomic status, *SES* Socioeconomic status

^a^ Q1 was the poorest and Q4 was the richest

^b^ Others include those who are divorced and widowed

**Table 4:** The relationship between contracting status and HRQOL stratified by chronic patients with different household income and educational attainment in rural Shandong, China, 2018

Characteristics	EQ-5D-3L values Coefficient (95%CI) ^a^	EQ-5D-3L dimension				
		**Mo** **AOR **^**b**^**(95%CI)**	**SC** **AOR (95%CI)**	**UA** **AOR (95%CI)**	**PD** **AOR (95%CI)**	**AD** **AOR (95%CI)**
**Household income**						
**Q1**						
Uncontracted	Ref	1.0	1.0	1.0	1.0	1.0
Contracted	0.116^**^ (0.039,0.194)	0.474^*^ (0.244,0.921)	0.465 (0.193,1.119)	0.57 (0.305,1.080)	0.430^**^ (0.240,0.772)	0.460^*^ (0.227,0.929)
**Q2**						
Uncontracted	Ref	1.0	1.0	1.0	1.0	1.0
Contracted	0.049 (-0.134,0.112)	0.442^*^ (0.226,0.863)	0.489 (0.199,1.201)	0.557 (0.293,1.060)	0.733 (0.433,1.241)	0.818 (0.428,1.560)
**Q3**						
Uncontracted	Ref	1.0	1.0	1.0	1.0	1.0
Contracted	0.013 (-0.051,0.076)	0.827 (0.398,1.697)	0.745 (0.263,2.109)	0.889 (0.444,1.778)	0.934 (0.525,1.661)	0.881 (0.409,1.900)
**Q4**						
Uncontracted	Ref	1.0	1.0	1.0	1.0	1.0
Contracted	-0.002 (-0.063,0.058)	0.602 (0.289,1.257)	0.708 (0.219,2.290)	0.286^**^ (0.116,0.698)	1.208 (0.723,2.021)	1.144 (0.599,2.186)
**Educational attainment**						
**Primary education or below**						
Uncontracted	Ref	1.0	1.0	1.0	1.0	1.0
Contracted	0.035 (-0.004,0.076)	0.586 (0.394,0.871)^**^	0.565 (0.331,0.965)^*^	0.525 (0.351,0.786)^**^	0.888 (0.631,1.249)	0.727 (0.491,1.077)
**Junior education**						
Uncontracted	Ref	1.0	1.0	1.0	1.0	1.0
Contracted	0.007 (-0.057,0.072)	0.627 (0.278,1.410)	0.570 (0.140, 2.318)	0.803 (0.368,1.752)	0.987 (0.579,1.685)	1.623 (0.776,3.392)
**Senior education or above**						
Uncontracted	Ref	1.0	1.0	1.0	1.0	1.0
Contracted	0.266 (0.119,0.413)^***^	0.088 (0.011,0.726)^*^	0.030 (0.001,1.318)	0.392 (0.099,1.546)	0.187 (0.064,0.545)^**^	0.124 (0.212,0.728)^*^

***Note***: *HRQOL* Health-related quality of life, *MO* Mobility, *SC* Self-care, *UA* Usual activity, *PD* Pain/discomfort, *AD* Anxiety/depression

*P*-values with statistical significance: ^*^P < 0.05, ^**^P < 0.01, ^***^P < 0.001

Adjusted for age, gender, marital status, sports participation, drinking status, multiple chronic diseases, family members

^a^ EQ-5D-3L utility values, Regression coefficient based on a Tobit regression fully adjusted for

^b^ Odds ratio based on a multivariate logistic regression fully adjusted for

## Discussion

The signing rate of family doctors with chronic diseases in rural areas of Shandong Province in the current study was approximately 28.9%. It was far lower than the target of 60% signing rate of key groups [13], and also lower than 67.6% signing rate in Eastern and Central Europe [28], which was consistent with previous studies [29, 30]. It indicated that there was still a long way to push forward the FDCS among the vulnerable groups of the population in rural China. This finding also implied a need for studies to explore the potential barriers in FDCS among rural patients with chronic conditions in China [31]. In addition, the signing rate among the high-income group (Q4; 34.4%) and middle-income group (Q2; 28.6% Q3; 31.3%) is higher than that among the low-income group (Q1; 23.3%). The signing rate of family doctors in patients with medium and high educational level was higher than that of patients with low educational level. The possible reason was that the patients with high income or high educational level might have better health awareness, tended to agree with advanced health service ideas, and had a higher demand of the FDCS.

The current study also demonstrated that FDCS was associated with HRQOL among patients with chronic diseases, and the contracted group reported higher HRQOL scores than the uncontracted group. This finding is consistent with previous studies. A study in Norway demonstrated that family doctors can improve health-related quality of life in older patients exposed to polypharmacy [32]. Another study in Turkey found that family doctors intervention improved clinical outcomes for diabetics [33]. Contracting with family doctors could improve disease awareness and treatment compliance in patients with chronic diseases such as hypertension, which was useful for controlling the anxiety level of patients [26, 34–36]. Poor medication compliance, mood depression and anxiety were found to be associated with poor quality of life. Consistent with other findings, we also found that the contracted group reported the lower proportion of problems in the five dimensions of HRQOL, which also proved that signing with a family doctor had a positive impact on both physiological and psychological well-being.

This study indicated that there was a significant difference in the association between contracting status and HRQOL across different income groups. Specifically, the results showed that only in the sub-high-income group, contracting status was correlated with HRQOL. One possible interpretation for this finding might be that in the low and sub-low-income groups, patients with chronic diseases have lower levels of health literacy, lower rates of going to medical institutions, and tend to self-diagnose [37]. The high-income group would take the initiative to seek medical treatment, whether they signed up or not [36]. However, sub-high-income patients were familiar with the contents of FDCS, and they had higher rates of going to medical institutions [37]. Family doctors could help them to obtain timely and effective chronic disease management services so as to improve their HRQOL [38].

Our findings also showed that the HRQOL of contracted patients was significantly different from the HRQOL of non-contracted patients in the high educational level, while there was no significant difference in HRQOL between patients with middle and low levels of education. An interpretation for this finding might be that patients with chronic diseases with high educational level could better understand the relevant policies of FDCS, had higher acceptance of health knowledge [21], and had higher utilization willingness of contracted services, so they had been in better management of chronic diseases, and thus had higher EQ-5D-3L utility value. Contracted patients with low educational level may lead to low utilization rate of contracted services due to insufficient awareness of the contract content and low health literacy [39, 40].

Interestingly, we found that the employment difference in the association between contracting status and EQ-5D-3L utility value was not significant. The possible reason was that the composition of urban occupation was complex, and there were differences in the working environment and medical resources among different types of occupations. However, the composition of rural occupation was simple, most of the residents were engaged in agriculture or forestry, and there was no significant difference in the working environment.

This study is the first to comprehensively explore the relationship between FDCS and HRQOL among Chinese rural patients with chronic diseases. It has several implications for policy-makers. Firstly, the government should focus on the HRQOL of rural patients with chronic diseases and increase the rate of signing family doctors. Based on the existing local health resources, the government should improve the service quality and enhance the residents' trust in the clinics. In view of the priority population, health management should be strengthened to meet personalized health needs, so as to improve the enthusiasm of patients to contract with family doctors. Secondly, our findings confirm the importance of accounting for SES when evaluating the effectiveness of FDCS, so the local government should develop a targeting signing plan for different subpopulation to reduce health inequalities.

Our study has several limitations. First, the cross-sectional data used in this study does not allow determining whether the associations detected reflect a causal relationship, and a longitudinal design is needed in the future study. Second, we only included patients with hypertension, diabetes, and heart disease in the study, the types of chronic diseases involved were limited, and the severity and duration of chronic diseases were not carefully divided, which would be gradually supplemented in the follow-up study. Third, only one member of a family was selected by multi-stage random sampling. The composition of samples of different characteristics of people might result in some bias of the results, which would be remedied in the follow-up study.

## Conclusions

This study demonstrated a significant association between FDCS and HRQOL among the patients with chronic diseases in rural China, and there were differences in this relationship among patients with different SES levels. Compared with non-contracted patients, contracted patients reported significantly higher HRQOL scores in low-income or high educational level patients. Therefore, policies should focus on improving the competence of family doctors and the signing rate of key groups. The government should take efforts to offer tailored contract service packages in line with residents’ socioeconomic status, so as to improve the quality of life of the elderly in rural areas.

## Supplementary Information

**Additional file 1: Supplementary Table 1**. Family role characteristics of participants stratified by age.

## Data Availability

Not applicable.

## References

[1] World Health Organization (2020). World health statistics 2020: monitoring health for the SDGs, sustainable development goals.

[2] World Bank (2011). Toward a healthy and harmonious life in China: stemming the rising tide of non-communicable diseases.

[3] Joanne EJ, Gauden G, Colin T, Robert B (2005). Chronic diseases 2: preventing chronic diseases: taking stepwise action. Lancet.

[4] Reynolds R, Dennis S, Hasan I, Slewa J, Chen W, Tian D (2018). A systematic review of chronic disease management interventions in primary care. BMC Fam Pract.

[5] Barbara S, Leiyu S, James M (2010). Contribution of primary care to health systems and health. Milbank Q.

[6] Pedersen KM, Andersen JS, Søndergaard J (2012). General practice and primary health care in Denmark. J Am Board Fam Med.

[7] Bindman AB, Majeed A (2003). Primary care in the united states: organisation of primary care in the united states. BMJ Brit Med J.

[8] Phillips RL, Bazemore AW, DeVoe JE (2015). A family medicine health technology strategy for achieving the triple aim for US health care. Fam Med.

[9] Pertusa-Martínez S (2006). General practitioners at the court of Queen Elizabeth II of England. Experience of a Spanish family doctor in the United Kingdom. Atencion Primaria.

[10] Comino MS, Krane S, Schelling J, Regife GV (2016). Differences and similarities of primary care in the german and spanish health care systems. Atención Primaria.

[11] Bowman MA, Neale AV (2013). Family physicians improve patient health care quality and outcomes. J Am Board Fam Med.

[12] Lam CL (2016). The role of the family doctor in the era of multi-disciplinary primary care. Fam Pract.

[13] National Health and Family Planning Commission (2016). Guideline on promoting family doctor contract service, vol. 1.

[14] The Centers for Disease Control and Prevention. Health-related Quality of Life. Retrieved October 31, 2018 from https://www.cdc.gov/hrqol/.

[15] McCaffrey N, Kaambwa B, Currow DC, Ratcliffe J (2016). Health-related quality of life measured using the EQ-5D-5L: South Australian population norms. Health Qual Life Outcomes.

[16] Jing Z, Li J, Wang Y, Yuan Y, Zhao D, Hao W, Yu C, Zhou C. Association of smoking status and health-related quality of life: difference among young, middle-aged, and older adults in Shandong, China. Qual Life Res. 2021;30(2):521–30. 10.1007/s11136-020-02645-9 32989682

[17] Grotto I, Huerta M, Sharabi Y (2008). Hypertension and socioeconomic status. Curr Opin Cardiol.

[18] Mielck A, Reitmeir P, Vogelmann M, Leidl R (2013). Impact of educational level on health-related quality of life (HRQL): results from Germany based on the EuroQol 5D (EQ-5D). Eur J Public Health.

[19] Hongmei L, Yuan G, Qi M, Anqi C, Aijun X (2019). Study on the impact of family doctor contracting service on health service utilization of chronic patients. Health Econ Res.

[20] Stafford M, Soljak M, Pledge V, Mindell J (2012). Socio-economic differences in the health-related quality of life impact of cardiovascular conditions. Eur J Public Health.

[21] Zhao Y, Lin J, Qiu Y, Yang Q, Wang X, Shang X, Xu X (2017). Demand and signing of general practitioner contract service among the urban elderly: a population-based analysis in Zhejiang Province, China. Int J Environ Res Public Health.

[22] Liu Z, Tan Y, Liang H, Gu Y, Wang X, Hao Y, Gu J, Hao C. Factors influencing residents' willingness to contract with general practitioners in Guangzhou, China, during the GP Policy Trial Phase: a cross-sectional study based on Andersen's behavioral model of health services use. Inquiry. 2019;56:46958019845484. 10.1177/0046958019845484 PMC6537300 31084420

[23] Szende A, Oppe M, Devlin N, eds. EQ-5D Value Sets: Inventory, Comparative Review and User Guide. EuroQol Group Monographs Vol. 2. New York: Springer; 2007.

[24] Kulkayeva G, Harun-Or-Rashid M, Yoshida Y, Tulebayev K, Sakamoto J (2012). Cardiovascular disease risk factors among rural Kazakh population. Nagoya J Med Sci.

[25] Falagas ME, Zarkadoulia EA, Pliatsika PA, Panos G (2008). Socioeconomic status (SES) as a determinant of adherence to treatment in HIV infected patients: a systematic review of the literature. Retrovirology.

[26] Institute of Medicine (US) Committee on Environmental Justice (1999). Toward environmental justice: research, education, and health policy needs.

[27] Winkleby MA, Jatulis DE, Frank E, Fortmann SP (1992). Socioeconomic status and health: how education, income, and occupation contribute to risk factors for cardiovascular disease. Am J Public Health.

[28] Kersnik J (2001). Determinants of customer satisfaction with the health care system, with the possibility to choose a personal physician and with a family doctor in a transition country. Health Policy.

[29] Aubin M, Vezina L, Verreault R, Fillion L, Hudon E, Lehmann F, Leduc Y, Bergeron R, Reinharz D, Morin D (2011). Family physician involvement in cancer care and lung cancer patient emotional distress and quality of life. Support Care Cancer.

[30] Zheng Q, Shi L, Pang T, Leung W (2021). Utilization of community health care centers and family doctor contracts services among community residents: a community-based analysis in Shenzhen, China. BMC Fam Pract.

[31] Yaoqin W, Hongmei L, Anqi C, et al. Study on signing status and influencing factors of the family doctor service for residents-taking Jiangsu Province as an example. Modern Prev Med. 2020;47(4):631–4. (Chinese)

[32] Romskaug R, Skovlund E, Straand J, Molden E, Kersten H, Pitkala KH, Lundqvist C, Wyller TB (2019). Effect of clinical geriatric assessments and collaborative medication reviews by geriatrician and family physician for improving health-related quality of life in home-dwelling older patients receiving Polypharmacy: a cluster randomized clinical trial. JAMA Intern Med.

[33] Göktas O, Gül ÖÖ, Ertürk E (2016). Changes in the management of type 2 diabetic patients in family medicine practices in the Bursa region. Prim Care Diabetes.

[34] Yong W (2018). Influence of family physician contract service on treatment compliance of patients with chronic diseases. Chronic Pathematology J.

[35] Cupples M, Heron N. What to do after cardiac rehabilitation programs: the role of the general practitioner in cardiovascular prevention. Monaldi Arch Chest Dis. 2016;86(1–2):755. 10.4081/monaldi.2016.755 27748471

[36] Piotrowska DE, Pędziński B, Jankowska D, Huzarska D, Charkiewicz AE, Szpak AS (2018). Socio-economic inequalities in the use of dental care in urban and rural areas in Poland. Ann Agric Environ Med.

[37] Long Y. Comparative analysis of different income families' medical service demands——a survey based on 2557 different income households in china. J Changchun Univ. 2010;20(011):93–96. (Chinese)

[38] Shuo C (2018). Research on basic health status of low-income population. Health Econ Res.

[39] Manxiu N, Pingping Y. Analysis on the rural elderly chronic patient’s seeking healthcare behavior and its influencing factors: Empirical analysis based on CHARLS Data. Chin J Health Policy. 2016;5:35–41. (Chinese)

[40] Sule SS, Ijadunola KT, Onayade AA, Fatusi AO, Soetan RO, Connell FA (2008). Utilization of primary health care facilities: lessons from a rural community in southwest Nigeria. Nigerian J Med J Natl Assoc Resident Doctors Nigeria.

