# Supplementary material for: Family doctor contract services and health-related quality of life among patients with chronic diseases in rural China: what is the role of socioeconomic status?
Source: Int J Equity Health. 2021 Aug 26;20:191. doi: 10.1186/s12939-021-01530-2 (PMC8394049; doi:10.1186/s12939-021-01530-2)
Supplement: Supplementary file 1 — Additional file 1: Supplementary Table 1. Family role characteristics of participants stratified by age. [file 12939_2021_1530_MOESM1_ESM.docx]

**Supplementary Table 1. Family role characteristics of participants stratified by age**

| Family role variable | N (%) | Age (%) | | | |
| --- | --- | --- | --- | --- | --- |
|  |  | ＜50 | 50-59 | 60-69 | ≥70 |
| The head of the household | 667 (55.1) | 53 (8.0) | 131 (19.6) | 277 (41.5) | 206 (30.9) |
| Spouse of the head of the household | 482 (39.8) | 58 (12.0) | 145 (30.1) | 209 (43.4) | 70 (14.5) |
| Others | 61 (5.1) | 7 (11.5) | 8 (13.1) | 18 (29.5) | 28 (45.9) |
